# School environment assessment tools to address behavioural risk factors of non-communicable diseases: A scoping review

**DOI:** 10.1016/j.pmedr.2018.01.014

**Published:** 2018-01-31

**Authors:** Kiran Saluja, Tina Rawal, Shalini Bassi, Soumyadeep Bhaumik, Ankur Singh, Min Hae Park, Sanjay Kinra, Monika Arora

**Affiliations:** a Health Promotion Division, Public Health Foundation of India, India; b Australian Research Centre for Population Oral Health (ARCPOH), Adelaide Dental School, The University of Adelaide, Australia; c Centre for Health Equity, Melbourne School of Population and Global Health, University of Melbourne, Australia; d Department of Health Services Research and Policy, Faculty of Public Health and Policy, London School of Hygiene and Tropical Medicine, United Kingdom; e Department of Non-Communicable Disease Epidemiology, Faculty of Epidemiology and Population Health, London School of Hygiene and Tropical Medicine, United Kingdom

**Keywords:** Scoping review, School environment assessment, Non-communicable disease, School health, Adolescents, Diet, Physical activity, Tobacco, Alcohol

## Abstract

We aimed to identify, describe and analyse school environment assessment (SEA) tools that address behavioural risk factors (unhealthy diet, physical inactivity, tobacco and alcohol consumption) for non-communicable diseases (NCD). We searched in MEDLINE and Web of Science, hand-searched reference lists and contacted experts. Basic characteristics, measures assessed and measurement properties (validity, reliability, usability) of identified tools were extracted. We narratively synthesized the data and used content analysis to develop a list of measures used in the SEA tools.

Twenty-four SEA tools were identified, mostly from developed countries. Out of these, 15 were questionnaire based, 8 were checklists or observation based tools and one tool used a combined checklist/observation based and telephonic questionnaire approach. Only 1 SEA tool had components related to all the four NCD risk factors, 2 SEA tools has assessed three NCD risk factors (diet/nutrition, physical activity, tobacco), 10 SEA tools has assessed two NCD risk factors (diet/nutrition and physical activity) and 11 SEA tools has assessed only one of the NCD risk factor. Several measures were used in the tools to assess the four NCD risk factors, but tobacco and alcohol was sparingly included. Measurement properties were reported for 14 tools.

The review provides a comprehensive list of measures used in SEA tools which could be a valuable resource to guide future development of such tools. A valid and reliable SEA tool which could simultaneously evaluate all NCD risk factors, that has been tested in different settings with varying resource availability is needed.

## Highlights

• 24 school environment assessment (SEA) tools were identified by the scoping review.
• Majority SEA tools assessed diet/nutrition and physical activity.
• Tobacco and alcohol was sparingly assessed in SEA tools.
• Comprehensive list of measures used in SEA tools is provided.
• Valid and reliable SEA tools to evaluate all NCD risk factors is needed.

## 1 Background

The shift from United Nation's Millennium Development Goals to the Sustainable Development Goals (SDG) reflects a paradigm shift in terms of strategies to reduce premature mortality due to non-communicable diseases (NCDs) (UN, 2016). Tobacco use, physical inactivity, unhealthy diet and harmful alcohol use are the key behavioural risk factors responsible for significant mortality and morbidity due to NCDs (GBD 2015 DALYs and HALE Collaborators, 2016). These behaviours become established in childhood and adolescence (WHO, 2009) and contribute to overweight/obesity, raised blood pressure, raised blood glucose and dyslipidaemia (Li et al., 2013), which are precursors to adult chronic diseases. The prevalence of NCDs in children and youth is also increasing with decreased age of onset of these diseases (WHO, 2014). The WHO's voluntary global NCD targets aim to achieve country specific reductions in NCD risk behaviours and halt the rise of obesity and diabetes among adolescents and adults (WHO, 2014). Achieving these goals could substantially prevent premature heart disease, premature stroke, type 2 diabetes and cancer (WHO, 2005). Therefore, it is necessary to establish healthy behaviours earlier in life to prevent NCDs throughout the life-course.

Schools are uniquely positioned as an ideal setting to promote and reinforce healthy behaviours among children and adolescents (Singh et al., 2017). However, the extent to which ‘school environments’ have become unhealthy in recent years is a cause of major concern in both developed and developing countries (Story et al., 2009), (Meenakshi et al., 2012). Studies have shown that inadequate school built environment and school-level policies may negatively impact body mass index, (Galvez et al., 2010), (James et al., 2012), (Williams et al., 2013), (Duncan et al., 2014) physical activity and dietary behaviours (Jaime and Lock, 2009) among children. Concurrently, studies have shown that school policies and curriculums can positively impact on behaviours related to NCD risk factors, such as reducing tobacco use, intention and susceptibility (Arora et al., 2011), promoting physical activity and healthy dietary habits (Saraf et al., 2015).

Building on the Ottawa Charter for Health Promotion (1986) and the Jakarta Declaration for Promoting Health (1997), WHO has launched the Global School Health Initiative to increase the number of health promoting schools worldwide (WHO, 2017a, WHO, 2017b). The recent Shanghai Declaration also reaffirms the stand as it calls for health being created ‘in the settings of everyday life’ (WHO, 2016). Periodic assessment of school environments and its impact on NCD risk behaviours is imperative to ensure that schools are health promoting and discouraging unhealthy behaviours. This requires appropriate school environment assessment (SEA) tools that can be culturally adapted and contextualized in different settings. In spite of interest in building better school environments to modulate behavioural risk factors, there exists no comprehensive review of SEA tools for the four key behavioural NCD risk factors. We aimed to identify, describe and analyse SEA tools that address behavioural risk factors for non-communicable diseases (i.e. unhealthy diet, physical inactivity, tobacco and alcohol consumption).

## 2 Methodology

### 2.1 Justification of study design

We chose a scoping review design over other evidence synthesis methodologies, to develop understanding of the extent, range and nature of school environment tools (Hilary and Lisa, 2007). Methodological quality assessment for individual studies was not conducted as the aim of the study was to identify the types of SEA tools and measures available, rather than to evaluate the quality of studies (Levac et al., 2010).

We conducted content analysis to analyse the SEA tools. Content analysis enables drawing of inferences by coding textual materials in a valid and replicable manner, by systematically evaluating documentary materials.

### 2.2 Criteria for including studies in the review

We conducted a scoping review of studies which have described tools to assess school environment specifically in relation to behavioural NCD risk factors (unhealthy diet, physical inactivity, tobacco and alcohol consumption). The detailed inclusion and exclusion criteria are given in Table 1.

**Table 1:** Eligibility criteria for inclusion and exclusion of school environment assessment tools.

Inclusion criteria	Exclusion criteria
1. School environment tools to specifically evaluate the environment related to behavioural NCD risk factors (i.e. unhealthy diet, physical inactivity, tobacco and alcohol consumption) in schools. School environment in this context refers to all school-level attributes which directly or indirectly influence NCD risk factors among children and adolescents. These include built environment of the schools and the formal or informal school level policies and activities which informs health behaviours and knowledge of NCD risk factors among children and school staff. 2. Published in English Language 3. Published on or after 1990.	1. Tools which have assessed educational environment, or school mental health. 2. Tools which have assessed environment in pre-schools, colleges (degree schools), or schools for especially abled individuals. 3. Tools exclusively assessing behaviours, knowledge, attitude and practices of children/school staff without assessing other determinants related to school activities or policies. 4. identified records in which neither the full tool nor the psychometric properties were retrieved.

### 2.3 Search methods for identification of studies

#### 2.3.1 Electronic searches

We searched two electronic databases MEDLINE [Ovid] and Web of Science for articles published from 1990 onwards (last searched on 4th January 2014). We developed a search strategy for MEDLINE by combining key concepts related to the study as follows:

• School environment
  [(school.mp. or exp Schools/ AND exp Environment/ or environment.mp.) OR school environment.mp. OR (school adj6 environment).mp. OR exp. Policy/ OR (school adj3 policy).mp. OR school health.mp. or exp. School Health Services/),
• Assessment and research tools
  evaluat$.mp. OR observ$.mp. OR measur$.mp. OR assess$.mp.
  instrument$1.mp. OR scale.mp. OR tools.mp. OR questionnaire.mp. or exp Questionnaires/
• NCD risk factors
  exp Obesity/ or obes$.mp. OR life style.mp. or exp. Life Style/OR diet$.tw. OR eat$.tw. OR nutrition$.tw. OR (physical adj1 activit$).tw. OR exercise.tw. OR play.tw. OR (tobacco or alcohol).mp.
• Children and adolescents
  (child$ or adolescent$).mp. NOT (infant or preschool OR adult$ or pregnan$).mp.

The above search strategy was tailored and adapted for Web of Science.

#### 2.3.2 Searching other sources

We hand searched the reference list of eligible articles found by other methods, and contacted authors of included studies and experts in the field (including personnel in education sector identified vide contacts of review authors and published articles) to identify relevant studies and grey literature.

### 2.4 Selection of studies

After removing duplicates using Endnote 6, two authors (KS and AS) independently screened all records based on the article titles to exclude obviously ineligible articles. Abstracts of records not excluded at this stage were independently assessed for eligibility by KS and AS. Full texts of all articles not excluded at the abstract-screening stage were obtained and independently reviewed by KS and TR to assess final inclusion as per eligibility criteria. Any disagreements, at any phase, were resolved by discussion to build consensus.

### 2.5 Retrieving the identified tools and their quality assessment

The SEA tools were obtained through the following methods when articles consistent with our eligibility criteria did not include tools in either full text or supplementary material:

• Searching the name of the tool and/or the study in Google Search engine
• Requesting the corresponding author for the complete school environment assessment tool through email

### 2.6 Data charting

Relevant data were charted from all eligible studies and tools using a pre-designed form. This form captured the basic study characteristics and measurement properties (validity, reliability, usability) of the tools. Data were extracted for sample characteristics, type of reliability testing, test results and authors conclusions when studies reported the validity and/or reliability of tools. This form was initially piloted for suitability on two tools by two independent researchers (KS and AS) and amended where necessary.

### 2.7 Data analysis

We categorized the identified records into three types based on the final availability of the complete SEA tool and measurement properties of the SEA tool:

A. identified records where both the full tool and measurement properties were retrieved by the review team
B. identified records where the full tool was retrieved but the measurement properties were not retrieved
C. identified records where the full tool was not retrieved but the measurement properties were retrieved

We sorted the charted data using content analysis for articles/tools in categories A and B above. Two researchers (KS and TR) independently screened each study tool to identify recurring items and grouped them together. A consensus initial coding classification scheme was developed by the two authors in the software Atlas.Ti 6.2 (qualitative data analysis software). Items which could not be coded as per the initial classification scheme were coded separately. Emerging codes were compared and assembled into tentative groups and these were further compared, reorganized, and merged to develop the final classification scheme. The final classification scheme was reapplied on all the tools.

We narratively synthesized the data on the following aspects:

1. major characteristics of all identified tools- including type of tool, type of respondent, number of questions asked/items observed, region, target population.
2. measurement properties of the tools classified as category A and C above - including inter-rater reliability, test–retest reliability and validity, if reported.

## 3 Result

### 3.1 Search results

**Fig. 1:**
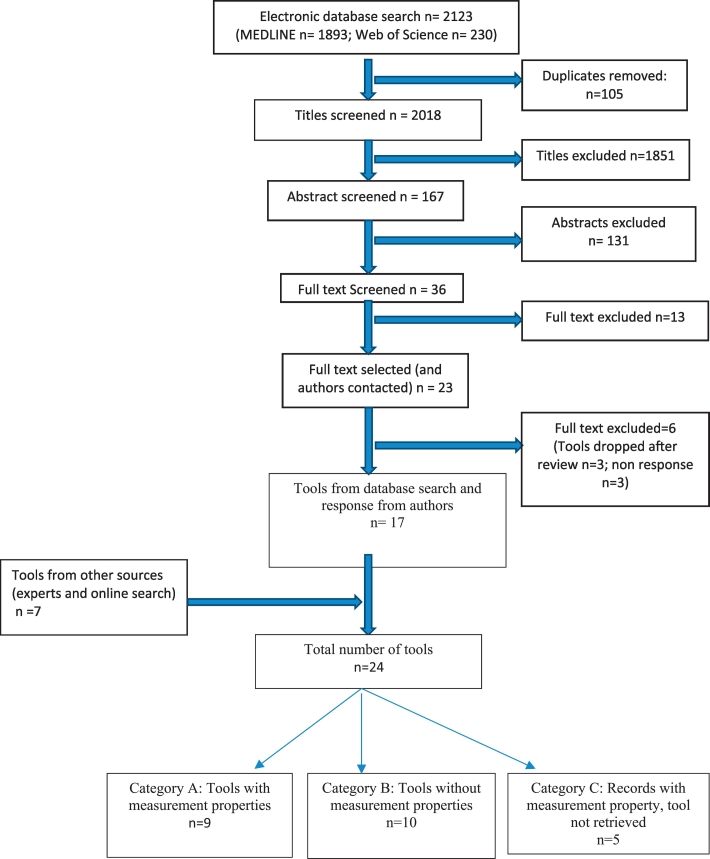
Flowchart for inclusion of studies.

In total, 2123 records were retrieved from the electronic databases search. Of these,105 duplicates were removed leading to screening of 2018 records. Additionally, we identified 7 tools by other methods (reference searching and expert contact). We finally included 24 tools based on our eligibility criteria. (Fig. 1**: flowchart for inclusion of studies**). We included 9 tools from 10 articles in category A (Kremer et al., 2006; Stigler et al., 2007; Erwin, 2008; Schwartz, 2008; Hearst et al., 2009; Jones et al., 2010; Krukowski et al., 2011; Brissette et al., 2013a, Brissette et al., 2013b; Lounsbery et al., 2013a, Lounsbery et al., 2013b; Nathan et al., 2013). There were 11 tools retrieved from 9 articles in category B (Harnack et al., 2000; NEAT, 2001; Mathews et al., 2008; CBSE, 2010; HSAT, 2011; Arora et al., 2012; CORD, 2012a, CORD, 2012b; Nazar, 2014; CDC, 2012), and 5 tools with only measurement properties of tools in category C (Brener et al., 2003; Finch et al., 2007; Bullock et al., 2010; Fisher et al., 2010; Wilson et al., 2013).

### 3.2 SEA tool characteristics

Characteristics of tools included in the review are presented in Table 2.

**Table 2:** Characteristics of included school environment assessment tools.

Sl. no	Name of tool/study (reference)	Country	Age/grade	Research tool	NCD risk behaviour/s	No. of items	Respondent
1	Speedy Audit^⁎^ (Jones et al., 2010)	UK	9–10 years (Grade 4) (Elementary)	Observation/checklist based	Physical activity	44	Research staff
2	Well SAT^#^ (Schwartz, 2008)	USA	Elementary to high school (All levels of schools)	Questionnaire	Diet/nutrition and physical activity	96	Research staff
3	School Cafeteria Nutrition Assessment^⁎^ (Krukowski et al., 2011)	USA	Pre-kindergarten through 12th grade (All levels of schools)	Observation/checklist based	Diet/nutrition	81	School staff
				Menu		93	
4	School Food Checklist^#^ (Kremer et al., 2006)	Australia	5–12 years (Elementary & middle)	Observation/checklist based	Diet/nutrition	20	Students
5	TREC-IDEA^⁎^ (Hearst et al., 2009)	USA	Middle schools	Observation/checklist based	Diet/nutrition	31	Research/non research staff
6	SPAPA^⁎,#^ (Lounsbery et al., 2013a, Lounsbery et al., 2013b)	USA	Elementary	Questionnaire	Physical activity	89	School staff, teachers (PE)
7	MYTRI^⁎;#^ (Stigler et al., 2007)	India	10–16 years	Questionnaire	Tobacco	84	Students
8	SEAT^⁎;#^ (Nathan et al., 2013)	Australia	5–18 years (All levels of schools)	Observation/checklist based+ telephonic interview	Diet/nutrition and physical activity	65	Teachers and principal
9	Pre-adol environmental access^⁎^ (Erwin, 2008)	USA	4th and 5th grade 9–12 years (Middle)	Observation/checklist based	Physical activity	12	Students
10	CATCH school health promotion^⁎;#^ (Texas Cord Project, 2012)	USA	1st–12th grade (All levels of schools)	Observation/checklist based	Diet/nutrition, physical activity and tobacco	8	Research staff
11	TX CORD^⁎;#^ (Texas Cord Project, 2012)	USA	1st–12th grade (All levels of schools)	Questionnaire	Diet/nutrition and physical activity	75; 113	Teachers; School staff
12	School environment audits (Be active eat well)^⁎;#^ (Mathews et al., 2008)	Australia	8–12 years (elementary & middle)	Questionnaire	Diet/nutrition and physical activity	53	School staff
13	School environment audits (It's your move) ^⁎;#^ (Mathews et al., 2008)	Australia	12–18 years (Middle and secondary and higher/senior secondary)	Questionnaire	Diet/nutrition and physical activity	25; 12;24	Principal, senior admin staff; Canteen manager; Teachers
14	Healthy school action tools (HSAT)^⁎;#^ (HSAT, 2011)	USA	Elementary–High School (All levels of schools)	Questionnaire	Diet/nutrition, physical activity and tobacco	365 (8 modules)	Research staff, school staff
15	Nutrition environment assessment tool (NEAT)* (NEAT, 2001)	USA	Elementary - high school (All levels of schools)	Questionnaire	Diet/nutrition	36	School staff
16	School Health Index^⁎;#^ (CDC, 2012)	USA	Elementary and high school (All levels of schools)	Questionnaire	Diet/nutrition, physical activity, tobacco and Alcohol	106 (8 modules)	Research staff, school staff, student
17	Nutrition policies and practices^⁎;#^ (Nazar, 2014)	India	1st–12th grade (All levels of schools)	Questionnaire	Diet/nutrition and physical activity	82	Teachers
18	Behavior and psychosocial survey^#^ (Arora et al., 2012)	India	8th and 10th grade (secondary level)	Questionnaire	Diet/nutrition and Physical activity	65	Students
19	La Carte food assessment^⁎^ (Harnack et al., 2000)	USA	Jr., Sr. and high schools (All levels of schools)	Observation/checklist based	Diet/nutrition	–	Food service managers
20	Food BEAMS^⁎#^ (Bullock et al., 2010)	USA	High schools	Computerized questionnaire	Diet/nutrition	NA	Research staff
21	SEHLS^#^ (Finch et al., 2007)	Australia	4–6 years (elementary)	Questionnaire	Diet/nutrition and physical activity	35	Students
22	Pedestrian environment data scan^⁎;#^ (Fisher et al., 2010)	USA	Not Reported	Observation/checklist based	Physical activity	115	Trained auditors (college students)
23	Eat well be active^⁎;#^ (Wilson et al., 2013)	Australia	5–18 years (All levels of schools)	Questionnaire	Diet/nutrition and physical activity	27; 15	Parents;Teachers
24	SHPPS^⁎;#^ (Brener et al., 2003)	USA	Elementary – High (All levels of schools)	Questionnaire	Diet/nutrition and physical activity	103	Students

Most SEA tools (14, 58.3%) were developed in the United States of America, followed by six (25%) in Australia, three (12.5%) in India and only one tool in the United Kingdom. Of all the identified SEA tools, 13 were published after 2009.

Of all the tools, 15 were questionnaire based (one was computerized), eight were checklists or observation based tools, and, one tool used a checklist/observation based method with a telephonic questionnaire.

Six SEA tools were administered to students (two observation/checklist type and four questionnaires) and 10 questionnaires were administered to the school staff (teachers, principal or administrative staff and the canteen/food managers). Four observation/checklists and two questionnaires were administered by research staff. Respondents of two questionnaires included both the school staff and the researcher.

12 (50%) SEA tools were developed for the use at all levels of schools (elementary, middle, secondary and higher/senior secondary), three SEA tools were for use only in elementary school level, two in middle level school only, one in secondary level, one in higher/senior secondary level only, two in elementary and middle level, one in middle and secondary level, one in middle, secondary and higher/senior secondary level. One SEA tool did not report the level of the school. Only one SEA tool had components related to all the four NCD risk factors (diet/nutrition, physical activity, tobacco and alcohol), two SEA tools assessed three NCD risk factors (diet/nutrition, physical activity, tobacco), 10 SEA tools assessed two NCD risk factors (diet/nutrition and physical activity), and 11 SEA tools assessed only one NCD risk factor (six SEA tools on diet/nutrition only; four SEA tools on physical activity only; one SEA tool on tobacco only).

### 3.3 Measures used in school environment assessment tool

We included 19 tools under category A and B for the content analysis. Emerging codes from the tools were categorized along the following dimensions: school policies (written/unwritten), school built environment, and interpersonal characteristics of the tools across the four NCD risk factors. Broadly, the key variables in SEA tools include school physical activity environment, school meal environment, nutrition and physical education at schools, tobacco and alcohol control policies, health assessment and community participation. Additionally, common items like provision of school-based health assessment and health and policy communication to students, parents and community, health programmes for parents, staff wellness, school health grants and school health improvement plans were also mentioned. The full details of the measures that have been used are given in Table 3.

**Table 3:** Measures used in school environment assessment tools to address major NCD risk factors.

School policies (written/unwritten)	School built environment	Inter-personal factors
Diet and nutrition		
**Nutrition education (NE) strategies, interventions & programmes** - NE for students, teachers/school staff, parents/community - Grade specific NE curriculum - Frequency and duration of NE classes - NE qualifications and NE trainings of teacher/school staff - Need assessment **Food/meals provided at schools** - Regulate sale of unhealthy food and beverages - Timings to access - Pricing, portion size, hygiene, food labelling and colour coding of food sold - Monitoring canteen environment, menu review and compliance to school policies and food safety standards **Lunchbox policies**: - Guidelines for parents, - Lunchbox monitoring by school staff - Restricting type of food and portion size brought from home **Advocate healthy diet and nutrition** - Access to food stores/outlets/vendors outside the school, during school hours - Programmes/provisions for events promoting healthy food advertising - School support to promote access to healthy food - Food as reward or punishment **Monitoring and evaluation of school food environment** - Ensuring healthy food served in school campus during class parties, school events, in canteens and school stores and fund raising	**Availability and promotion of unhealthy food** **Canteen/vending machines** - Availability and access - Type and price of food and beverages served, food hygiene and safety **School meals** - Provision, quality and menu cycle of school meals **Food vendors outside school** - Availability and access to food vendors outside school - Restricting or promoting healthy or unhealthy foods and beverages, portable drinking water, and healthy food advertising events **Signage in school campus**	Stakeholder's knowledge, perception and self-efficacy Awareness of school policies Role model for healthy eating (teachers/school staff, family/community) School staff's preparedness to respond food related emergencies School staff's motivation and skill to lead NE programmes Lunchbox monitoring by school staff and parents Student food preference Community/family support for healthy eating

Physical activity		
**Physical education (PE)** - PE for students, teachers/school staff, parents/community - Grade specific PE curriculum - Duration of PA and PE classes - Qualification and professional development of PE teachers **Physical activity (PA) strategies, interventions and programmes, resources** - Ensures safe access to schools - Promote walking & cycling around school - Regulate traffic congestion around schools - Use PA as reward/punishment - Prohibiting/exemption for PE/PA classes - PA safety standards - School play areas (access, utilization and inspection) - Provisions for enabling sport environment (sport equipments) and school support for active participation in inter and intra school sports events - School plans or goals to promote PA and physical fitness among students, school staff, parents/community	**School grounds and surroundings (around school)** - **A**vailability of safe play areas in and around school - Aesthetics, usage, quality and access to school grounds **PA resources in school/school sports environment** - Availability, access, quality and adequacy of sports equipment during and after **PE/PA classes and recess** **Signage in school campus**	Stakeholder's knowledge, perception and self-efficacy around physical activity Awareness of school policies Role model for active living (teachers/school staff, family/community) Involvement of teachers, school staff and parents to promote PA

Tobacco use		
- Provision of alcohol related education students/curriculum	- Availability of alcohol outlets (in and around school campus) - Signage to prohibit alcohol use (in and around school campus)	- Teachers/school staff, parents/community as role models - Stakeholder's knowledge and awareness of policies

Alcohol use		
- Provision of alcohol related education students/curriculum	- Availability of alcohol outlets (in and around school campus) - Signage to prohibit alcohol use (in and around school campus)	- Teachers/school staff, parents/community as role models - Stakeholder's knowledge and awareness of policies

### 3.4 Measurement properties of the included studies/tools

Measurement properties were reported for 14 tools. These included internal consistency (*n* = 3) inter-rater reliability (*n* = 7), test–retest reliability (*n* = 5), and validity of tools (*n* = 2). All tested the ability of tools to correctly measure the policy or built environment components of one or more NCD risk factors in schools. There was heterogeneity across the studies for assessment criteria, outcome variables, and effect measures. The measurement properties of the tools are summarized in Supplementary Appendix.

Internal consistency (Table A): Three studies reported internal consistency. One reported fair internal consistency for support for tobacco control policies (Cronbach's α = 0.92) but poor for knowledge about tobacco related public policies (Cronbach's α = 0.46) (Stigler et al., 2007). Another study that assessed children's exposure and teachers' knowledge, skills, and attitude, towards healthy diet and physical activity showed moderate to weak internal consistency of the questionnaire (Wilson et al., 2013). There was moderate internal consistency (Fisher et al., 2010) for overall walkability.

Inter-rater reliability (Table B): Five studies reported high inter-rater reliability of tools to assess diet and nutrition environment in schools (mean correlation = 0.95, SD = 0.07) (Kremer et al., 2006; Hearst et al., 2009; Bullock et al., 2010; Krukowski et al., 2011; Brissette et al., 2013a, Brissette et al., 2013b). High inter-rater reliability (mean % agreement =85.6, SD = 13.27; mean correlation = 0.86, SD = 0.01) was also observed for three studies (Fisher et al., 2010; Jones et al., 2010; Brissette et al., 2013a, Brissette et al., 2013b) that assessed physical education and physical activity environment in schools.

Test-retest reliability (Table C): Five studies showed weak to moderate test-retest reliability of SEA tools for physical activity and physical education policies (Lounsbery et al., 2013a, Lounsbery et al., 2013b), (Brener et al., 2003; Finch et al., 2007; Wilson et al., 2013) (Erwin, 2008). Moderate to high test-retest agreement was found for four tools assessing nutrition environment and related policies in schools (Brener et al., 2003; Finch et al., 2007; Lounsbery et al., 2013a, Lounsbery et al., 2013b; Wilson et al., 2013). The test-retest reliability was mostly poor for the tools assessing tobacco related school environment (Stigler et al., 2007; Brener et al., 2003). The test-retest period for all the included studies was between one to two weeks.

Validity (Table D): A 44 item audit tool (tested in 92 primary schools) reported good construct validity for physical activity opportunities in and around schools (Jones et al., 2010). A telephonic survey which was validated against an observation tool showed moderate to high validity test scores for canteen food and physical activity respectively (Nathan et al., 2013).

## 4 Discussion

The current study reviewed 24 tools including questionnaires and observation checklists applied to assess school environment and/or school policies specific to the four major NCD risk behaviours among school going children and adolescents. Only one SEA tool had components related to all the four NCD risk factors and only two had assessed three NCD risk factors. Tobacco use and harmful alcohol use are important NCD risk factors, more so for adolescent school children, when their use is initiated. Inclusion of alcohol and tobacco parameters in SEA tools is thus of critical importance and this has been sparsely been measured in SEA tools. Drawing conclusions about the quality, reliability and validity on most SEA tools was not possible as measurement properties were not reported.

Monitoring and evaluating school environment and policies is vital to optimize the availability of healthier food options in and around schools, promote healthy eating and physical activity in schools, restrict initiation and limit current tobacco and alcohol use by adolescents, and to identify major challenges in acquiring healthy behaviours among students (Sallis and Glanz, 2009). This is congruent with SDG 3.4 that aims to reduce premature mortality from NCDs by one third by 2030 (UN, 2016). The findings from this scoping review suggest a lack of comprehensive SEA tool to assess the school built environment and policies associated with the four key NCD risk behaviours, especially for the lower middle income countries (LMICs). This highlights the need to develop global checklists and standardised measures to evaluate school environments and school policies specific to NCD risk behaviours. However, global checklists and standardised measures need to be cautiously applied in the LMIC context considering the resource constraints when compared to high-income countries for adopting environmental changes across schools. As for example, in many LMIC countries are currently undergoing epidemiological transition and basic facilities like water and sanitation, which are taken for granted in high income countries might also need assessment along with those for NCDs as they might be accorded higher priority. A systematic review conducted to assess how government policies in LMICs influences actions related to diet or physical activity suggested mismatch between increasing prevalence of NCDs and their policy responses (Lachat et al., 2013). Creating and promoting health enabling environment at schools require interventions targeting schools' built environments as well as policies. The results of the current scoping review showed that more than half of the identified tools were designed to evaluate either the built environment or policies, not both.

Evidence from earlier studies showed that in addition to wider school environment and school staff, parents are also a key stakeholder in children's health related behaviours (Riggs et al., 2013). Parents thus have an important role to promote healthy lifestyle among children both in and outside schools and their role was evaluated in majority of tools. There is a need for greater recognition of this issue and inclusion in SEA tools.

### 4.1 Strengths and limitations

We have searched two electronic databases as well as used other methods in order to identify relevant studies. We however acknowledge that there might be additional SEA tools used by government departments or other agencies, which are present in reports or other formats that cannot be easily identified, although we have attempted to remove this bias by contacting some stakeholders. The exclusion of non-English tools and studies further limits inclusion of potentially relevant studies. We used independent data charting throughout to reduce any chance of reviewer bias. We attempted to get unpublished information on measurement properties of the tools by contacting authors, but we could not get the required information for all the tools. However, our rigorous approach has led to the best use of available data.

### 4.2 Implications of the study

The review collated multiple SEA tools that capture information key to reduction of NCD risk behaviours including unhealthy dietary pattern, physical inactivity, tobacco and alcohol use. We have provided a comprehensive list of tools, which practitioners and policy makers would find useful for selecting a tool fit for their purpose. It also provides information on the measurement properties of the tools, where available, and thus enables a quality comparison. We recommend that the measurement properties of such tools are evaluated during development and are reported in publications. Overall our review identifies the need to develop a comprehensive tool which evaluates all aspects of the school environment (including all major risk behaviours, both built environment and policy, and all key stakeholders), which has been validated and tested in different settings with varying resource availability.

To assess the school environment comprehensively a tool should ideally have all the measures and for all the four NCD risk factors as mentioned in Table 3 of our manuscript. We acknowledge that school environment assessment tools are context specific and resource dependent but the comprehensive list would serve as a useful guide for those developing school environment assessment tools. A comprehensive tool would thus include the assessment of following components:

• School health policies (national/school level; written/unwritten; including curriculum) with specific focus on prevention of all four NCD risk factors, tailored for each class/grade.
• Enabling school Built Environment (availability and accessibility of safe areas to promote physical activity and nutritious balanced diet and inhibiting tobacco and alcohol use) both within and around schools
• Interpersonal factors, including those influenced by peers as well as teachers and parents as role model

As an example, the Global School Health Survey- GSHS (WHO, 2015), is a widely applied tool but it focusses only on understanding the behaviours of school going students, and associated risk factors, without a focus on policies and built environments in schools. These elements are essential for children and adolescents to adopt and sustain healthy behaviours for prevention of NCDs. The GSHS could consider including an additional component to better understand the effectiveness and issues associated with school policies, monitor school built environments, and consequently enable policy makers and school authorities to promote healthy schools and children.

## 5 Conclusion

The review has identified available tools, and presented a comprehensive list of measures that can be used for the development of future SEA tools aimed at assessing school environments in relation to key behavioural risk factors for NCDs. Therefore, this study provides a valuable resource to guide further development of SEA tools and evaluations in future.

The following are the supplementary data related to this article.

Appendix Measurement Properties of included SEA tools. Appendix
