# Supplementary material for: School environment assessment tools to address behavioural risk factors of non-communicable diseases: A scoping review
Source: Prev Med Rep. 2018 Jan 31;10:1–8. doi: 10.1016/j.pmedr.2018.01.014 (PMC5984208; doi:10.1016/j.pmedr.2018.01.014)
Supplement: Appendix — Measurement Properties of included SEA tools. [file mmc1.docx]

| **Appendix : Measurement Properties of included SEA tools**  **Table A : Internal consistency** | | | | | | |
| --- | --- | --- | --- | --- | --- | --- |
| **S. No** | **Reference** | **Type** | **Variables tested** | **Internal consistency results (Cronbach’s α)** | **ICC** | **Author’s conclusion** |
| 1 | Stigler et al, 2007 | Scales | Knowledge of tobacco related public policies | Test (0.46); re-test (0.42) |  | The internal consistency of support was excellent but not for knowledge of public policies |
|  |  |  | Support for control policies | Test (0.92); re-test (0.93) |  |  |
| 2 | Wilson et al, 2013 | Teacher’s Questionnaire to assess teaching and teacher’s skills, attitudes, knowledge around healthy eating and Physical activity | **Child exposure** | |  | Internal consistency of the questionnaires was poor to moderate |
|  |  |  | Healthy eating | 0.61 | 0.86 (0.67 – 0.94) |  |
|  |  |  | Fruit and vegetables | 0.91 | 0.77 (0.52 – 0.90) |  |
|  |  |  | Physical activity | 0.58 | 0.67 (0.38 – 0.84) |  |
|  |  |  | **Teacher skills/attitudes** | |  |  |
|  |  |  | Healthy eating | 0.6 | 0.71 (0.40 – 0.88) |  |
|  |  |  | Fruit and vegetables | 0.11 | 0.61 (0.30 – 0.80) |  |
|  |  |  | Physical activity | 0.22 | 0.56 (0.11 – 0.82) |  |
|  |  |  | **School environment** |  | 0.67 (0.39 – 0.84) |  |
|  |  |  | **Teacher’s knowledge** | |  |  |
|  |  |  | Fruit |  | 0.42 (0.05 – 0.67) |  |
|  |  |  | Vegetables |  | 0.63 (0.33 – 0.81) |  |
|  |  |  | Screen time |  | 0.53 (0.20 – 0.76) |  |
|  |  |  | Physical activity |  | 0.81 (0.63 – 0.91) |  |
| 3 | Fisher et al, 2010 | Observation/ checklist based | **Overall Walkability** | 0.60 |  | Moderate internal consistency |

| **Table B: Inter-rater reliability** | | | | | | | | |
| --- | --- | --- | --- | --- | --- | --- | --- | --- |
|  |  |  |  |  |  |  |  |  |
| **S. No** | | **Ref.** | **Tool/ Method** | | **Variables tested** | | **Reliability results** | **Conclusion** |
| **Diet and nutrition** | | | | | | | | |
| 1 | | Brisette et al, 2013 | | Tool to assess elements of local wellness policy | Nutrition Education wellness promotion | Comprehensiveness | CC = 0.89 | Reliable and feasible tool for health and school agencies to use in assessing the policies included in Local wellness policy |
|  |  |  |  |  |  | Strength | CC = 1 |  |
|  |  |  |  |  | Nutrition Standards | Comprehensiveness | CC = 0.76 |  |
|  |  |  |  |  |  | Strength | CC = 0.84 |  |
| 2 | | Bullock et al, 2010 | | Tool to assess adherence to nutrition policies | Competitive Foods (researcher - researcher) | | ICC = 0.980, CI 0.934 - 0.994 | Reliable for both research and non- research professional |
|  |  |  |  |  | Competitive Beverage (researcher - researcher) | | ICC = 0.987, CI 0.963 - 0.996 |  |
|  |  |  |  |  | Competitive Foods (researcher - non - researcher) | | ICC = 0.972, CI 0.904 - 0.992 |  |
|  |  |  |  |  | Competitive Beverage (researcher - non - researcher) | | ICC = 0.977, CI 0.925 - 0.993 |  |
| 3 | | Hearst et al, 2009 | | Tool | Checklist | | Kappa = 0.99 | Reliable checklist approach satisfactory in case of limited resources |
|  |  |  |  |  | Inventory | | ICC = 0.9974 |  |
|  |  |  |  | Method | Comparing both methods to rank level of healthy foods available | | Wilkoxon Rank sum = 32.5, p = 0.62 |  |
| 4 | | Krukowski et al, 2011 | | Tool | Menu | Overall | ICC = 0.98 | High inter-rater reliability |
|  |  |  |  |  |  | Fruits | ICC = 0.98 |  |
|  |  |  |  |  |  | Vegetables | ICC = 0.99 |  |
|  |  |  |  |  |  | Grains | ICC = 0.98 |  |
|  |  |  |  |  |  | Side dishes | ICC = 0.38 |  |
|  |  |  |  |  |  | Entrees | ICC = 0.97 |  |
|  |  |  |  |  |  | Chips | ICC = 0.87 |  |
|  |  |  |  |  |  | Deserts | ICC = 0.95 |  |
|  | |  | |  | Observation | Overall | ICC = 0.94 |  |
|  |  |  |  |  |  | Fruits | ICC = 0.79 |  |
|  |  |  |  |  |  | Vegetables | ICC = 0.95 |  |
|  |  |  |  |  |  | Grains | ICC = 0.86 |  |
|  |  |  |  |  |  | Side dishes | ICC = 0.62 |  |
|  |  |  |  |  |  | Entrees | ICC = 0.92 |  |
|  |  |  |  |  |  | Chips | ICC = 0.41 |  |
|  |  |  |  |  |  | Deserts | ICC = 0.95 |  |
|  |  |  |  |  |  | A la carte | ICC = 0.79 |  |
|  |  |  |  |  |  | Beverages | ICC = 0.93 |  |
|  |  |  |  |  |  | Produce | % agreement = 94 |  |
|  |  |  |  |  |  | Side dishes | % agreement = 91 |  |
|  |  |  |  |  |  | Desert | % agreement = 91 |  |
| 5 | | Kremer et al, 2006 | | Tool - checklist | Lunches |  | Kappa 0.51 |  |
|  |  |  | | Method - Checklist vs weighted records |  |  | 0.77, p<0.01 |  |
| **Physical activity** | | | | | | | | |
| 1 | Jones et al, 2010 | | Tool | | Walking provision | Items = 5 | % agreement = 76.22 ; Kappa = 0.41 | Acceptable reliability and good construct validity |
|  |  |  |  |  | Cycling provision | Items = 9 | % agreement = 94.99; Kappa = 0.57 |  |
|  |  |  |  |  | Sports and play facility provision | Items = 16 | % agreement = 98.15; Kappa = 0.72 |  |
|  |  |  |  |  | Other facility provision | Items = 8 | % agreement = 92.71; Kappa = 0.36 |  |
|  |  |  |  |  | Design of the school grounds | Items = 6 | % agreement = 98.06 |  |
|  |  |  |  |  | Aesthetics | Items = 9 | % agreement = 98.83; Kappa = 0.07 |  |
|  |  |  |  |  | School Physical activity score | Items = 29 | % agreement = 98.88; Kappa = 0.48 |  |
| 2 | Fisher et al, 2010 | | Pedestrian environment Audit | | Walkability overall |  | % agreement = 86.2; Kappa = 0.72 |  |
| 3 | Brisette et al, 2013 | | Tool to assess policy | | Physical Education and Physical activity | Comprehensiveness of elements of LWP | CC = 0.85 | High inter-rater reliability |
|  |  |  |  |  |  | Strength of elements of LWP | CC = 0.87 |  |

| **Table C: Test-Retest reliability** | | | |  |  |  |  |  |  |
| --- | --- | --- | --- | --- | --- | --- | --- | --- | --- |
| S. No | Author |  | Test-retest period | Variables tested | Reliability results | Author’s conclusion |  |  |  |
| **Physical Activity** | | | | | | |  |  |  |
| 1 | Erwin et al, 2008 | Tool | 7- 10 days | **School Environment** |  |  |  |  |  |
|  |  |  |  | Blacktop playground | Kappa = 0.2 | Suitable tool for examining children’s physical activity supports and inhibitors |  |  |  |
|  |  |  |  | Grassy playground | Kappa = 0.74 |  |  |  |  |
|  |  |  |  | Gymnasium or cafeteria | Kappa = 0.32 |  |  |  |  |
|  |  |  |  | Intramurals | Kappa = 0.41 |  |  |  |  |
|  |  |  |  | Jungle gym (swings, monkey bars, slides) | Kappa = 0.3 |  |  |  |  |
|  |  |  |  | Physical education (PE) | Kappa = 0.52 |  |  |  |  |
|  |  |  |  | Playing fields (soccer,football, softball) | Kappa = 0.72 |  |  |  |  |
|  |  |  |  | Running track | Kappa = 0.6 |  |  |  |  |
|  |  |  |  | School sports teams | Kappa = 0.75 |  |  |  |  |
|  |  |  |  | Sport or exercise equipment | Kappa = 0.34 |  |  |  |  |
|  |  |  |  | School swimming pool | Kappa = -0.04 |  |  |  |  |
|  |  |  |  | Tennis courts | Kappa = 0.38 |  |  |  |  |
| 2 | Lounsbery et al, 2013 | Tool | 14 days | **Physical Education : Availability of existing written policies** | | Items are reliable and useful in assessing PA policies in elementary schools |  |  |  |
|  |  |  |  | District policy requiring schools to follow specific PE standards | Kappa = 0.64; % agreement = 77 |  |  |  |  |
|  |  |  |  | School policy requiring program to follow specific PE standards | Kappa = 0.73; % agreement = 84 |  |  |  |  |
|  |  |  |  | District policy requiring number of PE minutes or days per week | Kappa = 0.55; % agreement = 71 |  |  |  |  |
|  |  |  |  | School policy requiring number of PE minutes or days per week | Kappa = 0.49; % agreement = 71 |  |  |  |  |
|  |  |  |  | District policy specifying maximum student-to-teacher ratio for PE | Kappa = 0.35; % agreement = 67 |  |  |  |  |
|  |  |  |  | School policy specifying maximum student-to-teacher ratio for PE | Kappa = 0.51; % agreement = 79 |  |  |  |  |
|  |  |  |  | District policy requiring annual PE program evaluation | Kappa = 0.54; % agreement = 83 |  |  |  |  |
|  |  |  |  | School policy requiring annual PE program evaluation | Kappa = 0.14; % agreement = 77 |  |  |  |  |
|  |  |  |  | Requirement for PE teachers to attend staff development yearly | Kappa = 0.37; % agreement = 74 |  |  |  |  |
|  |  |  |  | **Physical Education : PE content, curriculum, and delivery** | | |  |  |  |
|  |  |  |  | Provision of financial support for professional development | % agreement = 83 |  |  |  |  |
|  |  |  |  | Provision of student assessment/evaluation plans | Kappa = 0.5;  % agreement = 67 |  |  |  |  |
|  |  |  |  | Requirements for use of a specific curriculum | % agreement = 79 |  |  |  |  |
|  |  |  |  | Using PA for disciplinary purposes | % agreement = 87 |  |  |  |  |
|  |  |  |  | Withholding students from PE for academic reasons | % agreement = 71 |  |  |  |  |
|  |  |  |  | Competing demands for PE space | Kappa = 0.51;  % agreement = 68 |  |  |  |  |
|  |  |  |  | Availability of PE budget | Kappa = 0.72;  % agreement = 84 |  |  |  |  |
|  |  |  |  | PE teacher involvement in budget decisions | Kappa = 0.52;  % agreement = 66 |  |  |  |  |
|  |  |  |  | **Continuous items** | | |  |  |  |
|  |  |  |  | Number of PE classes per week | CC= 0.96 |  |  |  |  |
|  |  |  |  | Total minutes of PE per week | CC = 0.7 |  |  |  |  |
|  |  |  |  | Number of students per PE class | CC = 0.94 |  |  |  |  |
|  |  |  |  | Student-to-licensed teacher ratio in PE classes | CC = 0.76 |  |  |  |  |
|  |  |  |  | Actual minutes in PE setting during class time | CC = 0.51 |  |  |  |  |
|  |  |  |  | Total minutes of recess per day | CC = 0.72 |  |  |  |  |
| 3 | Finch et al, 2007 | Tool | 7 days | How much time do you usually spend each day on the computer playing video games? | Kappa = 0.59,CI 0.48- 0.68 | Test-retest reliability was moderate to high |  |  |  |
|  |  |  |  | On week days how much time do you usually spend watching television (TV)? | Kappa = 0.51,CI 0.38 - 0.64 |  |  |  |  |
|  |  |  |  | How many times a week do you usually eat your meal at night in front of the television (TV)? | Kappa = 0.63,CI 0.56 - 0.71 |  |  |  |  |
|  |  |  |  | How much time do you usually spend each week playing games or doing activities that make you run around or huff and puff? | Kappa = 0.63,CI 0.53- 0.73 |  |  |  |  |
|  |  |  |  | What do you usually do at recess? | Kappa = 0.71,  CI 0.62- 0.79 |  |  |  |  |
| 4 | Wilson et al, 2013 | Tool - questionnaire | 2 weeks | **Child exposure** | | Test-retest reliability was moderate to high |  |  |  |
|  |  |  |  | Physical activity | ICC = 0.67,  CI 0.38 - 0.84 |  |  |  |  |
|  |  |  |  | **Teacher skills/attitudes** |  |  |  |  |  |
|  |  |  |  | Physical activity | ICC = 0.56,  CI 0.11- 0.82 |  |  |  |  |
|  |  |  |  | **Teacher knowledge** |  |  |  |  |  |
|  |  |  |  | Screen time | ICC =0.53 ,  CI 0.20 - 0.76 |  |  |  |  |
|  |  |  |  | Physical activity | ICC = 0.81,CI 0.63 - 0.91 |  |  |  |  |
| 5 | Brener, 2003 | Questionnaire | 10 and 20 days | **Physical Education**  Availability of weight training, Dodgeball/bombardment | Kappa =79.1  Kappa =76.4 | Some questions demonstrated poor reliability, but most exhibited moderate or substantial reliability, and some exhibited almost perfect reliability |  |  |  |
|  |  |  |  | Availability of Physical Education (PE) Teachers | Kappa =76.3 |  |  |  |  |
|  |  |  |  | Use of physical activities such as laps or push-ups to punish students for bad behaviour in PE | Kappa =71.5 |  |  |  |  |
|  |  |  |  | Students at your school taught dance activities | Kappa =71.3 |  |  |  |  |
|  |  |  |  | School’s physical activity and athletic facilities for community sponsored sports teams | Kappa =68.7 |  |  |  |  |
|  |  |  |  | Opportunities for students to participate in intramural activities or physical activity clubs | Kappa =66.0 |  |  |  |  |
|  |  |  |  | Availability of written PE curriculum | Kappa =58.4 |  |  |  |  |
|  |  |  |  | Opportunities for students to develop individualized physical activity plans | Kappa =53.4 |  |  |  |  |
|  |  |  |  | Monitoring progress toward reaching goals in an individualized physical activity plan | Kappa =51.0 |  |  |  |  |
|  |  |  |  | Students spend time on gymnastics; basketball; walking, jogging, or running; student-designed games | PC=0.67; PC=0.67; PC=0.62; PC=0.59 |  |  |  |  |
| **Diet and Nutrition** | | | | | | |  |  |  |
|  |  |  |  | How much pocket money are you usually given each week snacks and drinks? ?? | Kappa = 0.57,CI 0.46 - 0.67 |  |  |  |  |
| 1 | Finch et al, 2007 |  |  | Do you ever buy food from the school canteen? | Kappa = 0.53,CI 0.279-0.78 |  |  |  |  |
|  |  |  |  | What time of day do you usually buy food from the school canteen? | Kappa = 0.54,CI 0.45- 0.63 |  |  |  |  |
|  |  |  |  | How often do you buy food from the school canteen? | Kappa = 0.57,CI 0.48 -0.66 |  |  |  |  |
|  |  |  |  | Do you usually eat breakfast on school mornings? | Kappa = 0.58,CI 0.37 -0.79 |  |  |  |  |
|  |  |  |  | Do you usually eat recess when you are at school? | Kappa = 0.18,CI -0.07- 0.43 |  |  |  |  |
|  |  |  |  | Do you ever buy recess from the school canteen? | Kappa = 0.68,CI 0.58- 0.78 |  |  |  |  |
|  |  |  |  | Do you usually eat lunch when you are at school? | Kappa = 0.35,CI 0.08- 0.63 |  |  |  |  |
|  |  |  |  | How often do you buy lunch from the school canteen? | Kappa = 0.52,CI 0.34- 0.70 |  |  |  |  |
|  |  |  |  | Do you ever buy food or drinks from a vending machine that is in your school? | Kappa = 0.36,CI 0.19- 0.53 |  |  |  |  |
|  |  |  |  | Do you ever buy food or drinks from places outside school grounds during school time? | Kappa = 0.48,CI 0.26 - 0.69 |  |  |  |  |
| 2 | Lounsbery et al, 2013 | Tool | 14 days | **Recess items : Availability of existing written policies** |  |  |  |  |  |
|  |  |  |  | District policy specifying minutes per day for recess | Kappa = 0.61; % agreement = 74 | Test-retest reliability was mostly moderate |  |  |  |
|  |  |  |  | School policy specifying minutes per day for recess | Kappa = 0.61; % agreement = 74 |  |  |  |  |
|  |  |  |  | District policy requiring organized activities during recess | Kappa = 0.73; % agreement = 90 |  |  |  |  |
|  |  |  |  | School policy requiring organized activities during recess | Kappa = 0.48; % agreement = 84 |  |  |  |  |
|  |  |  |  | District policy requiring training for recess supervisors | Kappa = 0.67; % agreement = 84 |  |  |  |  |
|  |  |  |  | School policy requiring training for recess supervisors | Kappa = 0.63; % agreement = 84 |  |  |  |  |
|  |  |  |  | District policy specifying student-to-supervisor ratio during recess | Kappa = 0.75; % agreement = 84 |  |  |  |  |
|  |  |  |  | School policy specifying student-to-supervisor ratio during recess | Kappa = 0.76; % agreement = 87 |  |  |  |  |
|  |  |  |  | Daily provision of recess | % agreement = 90 |  |  |  |  |
|  |  |  |  | **Recess items : Recess supervision** |  |  |  |  |  |
|  |  |  |  | Conduct organized activities | Kappa = 0.78 ; % agreement = 97 |  |  |  |  |
|  |  |  |  | Supervisor encouragement of PA | % agreement = 77 |  |  |  |  |
|  |  |  |  | Posting of recess rules | Kappa = 0.81; % agreement = 90 |  |  |  |  |
|  |  |  |  | Recess rules taught to students | Kappa = 0.46; % agreement = 90 |  |  |  |  |
|  |  |  |  | Training of recess supervisors | Kappa = 0.48; % agreement = 84 |  |  |  |  |
|  |  |  |  | **Recess items : Access to recess** |  |  |  |  |  |
|  |  |  |  | Permission for students to stay indoors | Kappa = 0.33; % agreement = 71 |  |  |  |  |
|  |  |  |  | Activity opportunities during inclement weather | Kappa = 0.58; % agreement = 80 |  |  |  |  |
|  |  |  |  | Withholding recess for academic reasons | Kappa = 0.79; % agreement = 93 |  |  |  |  |
|  |  |  |  | Withholding recess for disciplinary reasons | Kappa = 0.77; % agreement = 93 |  |  |  |  |
|  |  |  |  | **Recess items : Recess equipment** |  |  |  |  |  |
|  |  |  |  | Budget for recess equipment/supplies | Kappa = 0.57; % agreement = 74 |  |  |  |  |
|  |  |  |  | Loose equipment available during recess | % agreement = 93 |  |  |  |  |
| 3 | Wilson et al, 2013 | Tool - questionnaire | 2 weeks | **Child exposure** |  |  |  |  |  |
|  |  |  |  | Healthy eating | ICC = 0.86, | tool is moderately reliable tools for simultaneously assessing child intakes, environments, attitudes, and knowledge associated with healthy eating and physical activity in schools |  |  |  |
|  |  |  |  | Fruit and vegetables | ICC = 0.77 ,CI 0.52 - 0.90 |  |  |  |  |
|  |  |  |  | **Teacher skills/attitudes** |  |  |  |  |  |
|  |  |  |  | Healthy eating | ICC = 0.71,CI 0.40 - 0.88 |  |  |  |  |
|  |  |  |  | Fruit and vegetables | ICC = 0.61,CI 0.30 - 0.80 |  |  |  |  |
|  |  |  |  | **Teacher knowledge** |  |  |  |  |  |
|  |  |  |  | Fruit | ICC = 0.42,CI 0.05 - 0.67 |  |  |  |  |
|  |  |  |  | Vegetable | ICC = 0.63 , CI 0.33 - 0.81 |  |  |  |  |
| 4 | Brener, 2003 | Questionnaire | 10 and 20 days | Does your school have a committee that includes students who provide suggestions for the school food service program? | Kappa=63.2 | some questions demonstrated poor reliability, but most exhibited moderate or substantial reliability, and some exhibited almost perfect reliability |  |  |  |
|  |  |  |  | Availability (weekly) of fruit in school | Kappa=62.1 |  |  |  |  |
|  |  |  |  | Frequency of low-fat recipe in past 30 days.  Never; Rarely; Sometimes; Almost always or always | Kappa=40.2 |  |  |  |  |
|  |  |  |  | Use of fat trimmed meat or lean meat in past 30 days | Kappa=38.6 |  |  |  |  |
|  |  |  |  | Availability of salt if students ask for it | Kappa=88.0 |  |  |  |  |
|  |  |  |  | Policy on prohibiting cigarette smoking by faculty and staff during school- related activity | Kappa=64.3 |  |  |  |  |
|  |  |  |  | Are any students at your school prohibited from wearing tobacco brand-name apparel or carrying merchandise that displays tobacco company names, logos, or cartoon characters | Kappa=53.4 |  |  |  |  |
|  |  |  |  | Policy specifically prohibit cigarette smoking by students outside or on school grounds; by visitors | Kappa=49.3  Kappa=47.1 |  |  |  |  |
| **Tobacco** | | | | | | |  |  |  |
| 1 | Hriday, University of Texas (Stigler et al, 2007) | Tools - scales |  | Knowledge of public policies | CC = 0.28 | The test-retest reliability of most scales was poor |  |  |  |
|  |  | Tools - scales |  | Support for control policies | CC =0.39 |  |  |  |  |
|  |  | Tools – items |  | Item 9 (Tobacco offers, chewing) | CC =0.49 |  |  |  |  |
|  |  | Tools – items |  | Item 10 (Tobacco offers, chewing) | CC =0.47 |  |  |  |  |
|  |  | Tools – items |  | Item 27 (Tobacco offers, cigarette) | CC =0.58 |  |  |  |  |
|  |  | Tools – items |  | Item 28 (Tobacco offers, cigarette) | CC =0.45 |  |  |  |  |
|  |  | Tools – items |  | Item 72 (Access to first tobacco product) | CC =0.11  (CI 0.67 - 0.94) |  |  |  |  |
| 2 | Brener, 2003 | Questionnaire | 10 and 20 days | **Tobacco and alcohol use prevention**  Service staff provide tobacco use cessation | Kappa=34.5 | The test-retest reliability of most scales was moderate to poor |  |  |  |
|  |  |  |  | Staff development on tobacco use prevention | Kappa=35.8 |  |  |  |  |
|  |  |  |  | Instruction on tobacco use prevention for students  Time spend teaching about tobacco use prevention | Kappa=49.2  Kappa=0.60 |  |  |  |  |
|  |  |  |  | Availability of alcohol and other drug use treatment | Kappa=54.6 |  |  |  |  |

**Table D: Validity**

| **S. No** | **Reference** | **Type** | **Criterion method** | **Variables tested** | **Validity results** | | | **Author’s conclusion** |
| --- | --- | --- | --- | --- | --- | --- | --- | --- |
|  |  |  |  |  | **Agreement** | **Kappa** | **t test (p value)** |  |
| 1 | Jones et al, 2010 | Tool –Constr-uct validity | Observations validated against MVPA scores of students | Walking provision |  |  | p<0.001 | Good construct validity |
|  |  |  |  | Cycling provision |  |  | p<0.001 |  |
|  |  |  |  | Sports and play facility provision |  |  | 0.034 |  |
|  |  |  |  | Other facility provision |  |  | 0.629 |  |
|  |  |  |  | Design of the school grounds |  |  | 0.093 |  |
|  |  |  |  | Aesthetics |  |  | 0.522 |  |
|  |  |  |  | School Physical activity score |  |  | 0.818 |  |
| 2 | Nathan et al, 2013 | Tool | Telephone survey tool validated against observation | **Food sold in canteen** | | | | |
|  |  |  |  | Fruits | 79 | 0.57 (0.26, 0.79 |  | Valid tool for assessing aspects of the school food and physical activity environment |
|  |  |  |  | Vegetables | 57 | −0.06 (−0.26, 0.14) |  |  |
|  |  |  |  | Water | 88 | 0.76 (0.49, 0.92 |  |  |
|  |  |  |  | Regular soft drinks | 76 | 0.52 (0.21, 0.76) |  |  |
|  |  |  |  | Diet soft drinks | 74 | 0.48 (0.16, 0.72) |  |  |
|  |  |  |  | Fruit juice | 90 | 0.81 (0.55, 0.95 |  |  |
|  |  |  |  | Other sweetened drinks | 69 | 0.38 (0.11, 0.66) |  |  |
|  |  |  |  | Confectionary | 62 | 0.16 (−0.1, 0.43) |  |  |
|  |  |  |  | Sweet and savory biscuits | 52 | 0.02 (−0.28, 0.32) |  |  |
|  |  |  |  | Potato crisps | 55 | 0.04 (−0.25, 0.34) |  |  |
|  |  |  |  | Deep fried foods | 69 | 0 (0, 0) |  |  |
|  |  |  |  | Ice creams covered in chocolate | 79 | 0.57 (0.26, 0.79 |  |  |
|  |  |  |  | Other ice creams | 88 | 0.76 (0.49, 0.92) |  |  |
|  |  |  |  | Food sold in fundraising |  |  |  |  |
|  |  |  |  | Fruit | 81 | 0.62 (0.32, 0.83 |  |  |
|  |  |  |  | Vegetables | 79 | 0.57 (0.26, 0.79 |  |  |
|  |  |  |  | Water | 88 | 0.76 (0.49, 0.92) |  |  |
|  |  |  |  | Regular soft drinks | 81 | 0.62 (0.32, 0.83) |  |  |
|  |  |  |  | Diet soft drinks | 88 | 0.76 (0.49, 0.92 |  |  |
|  |  |  |  | Fruit juice | 83 | 0.67 (0.37, 0.86 |  |  |
|  |  |  |  | Other sweetened drinks | 88 | 0.76 (0.49, 0.92 |  |  |
|  |  |  |  | Confectionary | 67 | 0.06 (−0.21, 0.33) |  |  |
|  |  |  |  | Sweet and savoury biscuits | 67 | −0.02 (−0.26, 0.22) |  |  |
|  |  |  |  | Potato crisps | 90 | 0.81 (0.55, 0.95 |  |  |
|  |  |  |  | Deep fried foods | 95 | 0.90 (0.68, 0.99 |  |  |
|  |  |  |  | Ice creams covered in chocolate | 93 | 0.86 (0.61, 0.97 |  |  |
|  |  |  |  | Other ice creams | 90 | 0.81 (0.55, 0.95 |  |  |
|  |  |  |  | **Physical activity facilities accessible at recess and lunch** | | | |  |
|  |  |  |  | Large asphalt areas | 86 | 0.71 (0.43, 0.89 |  |  |
|  |  |  |  | Large playing fields | 81 | 0.62 (0.32, 0.83 |  |  |
|  |  |  |  | Indoor activity spaces | 67 | 0.01 (−0.29, 0.32) |  |  |
|  |  |  |  | Playground markings | 90 | 0.81 (0.55, 0.95) |  |  |
|  |  |  |  | Fixed playground equipment | 74 | 0.48 (0.16, 0.72 |  |  |
|  |  |  |  | School sports equipment | 57 | −0.02 (−0.2, 0.15) |  |  |
|  |  |  |  | **Organized Physical Activity** | | | |  |
|  |  |  |  | Organized physical activity | 100 | 1 (1, 1) |  |  |
|  |  |  |  | Do teachers join in | 100 | 1 (1, 1) |  |  |
|  |  |  |  | Students access to small screen recreation |  |  |  |  |
|  |  |  |  | During Recess/Lunch | 37 | 0 (−0.12, 0.12) |  |  |
|  |  |  |  | Before school | 59 | 0.02 (−0.28, 0.31) |  |  |
|  |  |  |  | During wet weather sport | 73 | 0.46 (0.14, 0.72 |  |  |
|  |  |  |  | School Sport |  |  |  |  |
|  |  |  |  | K-2 have sport | 83 | 0.67 (0.37, 0.86 |  |  |
|  |  |  |  | 3-6 have sport | 90 | 0.81 (0.55, 0.95 |  |  |
